# MIKROBE: a feasibility study for a randomised controlled trial of one-stage or two-stage surgery for prosthetic knee infection

**DOI:** 10.1186/s40814-025-01634-4

**Published:** 2025-04-16

**Authors:** Rohini Terry, Sarah Dean, Patrick Hourigan, Hugh Ben Waterson, Vikki Wylde, Natalie Carpenter, Bethany Whale, Roy J. Powell, Polly Tarrant, Antonieta Medina-Lara, Abtin Alvand, Andrew D. Toms

**Affiliations:** 1 University of Exeter Medical School, St Luke’S Campus, Heavitree Road, Exeter, EX1 2LU UK; 2 Exeter Knee Reconstruction Unit, Princess Elizabeth Orthopaedic Centre, Royal Devon University Hospital NHS Trust, Barrack Road, Exeter, EX2 5DW UK; 3 Musculoskeletal Research Unit, NIHR Bristol Biomedical Research Centre, University Hospitals Bristol and Weston NHS Foundation Trust, Bristol Medical School, University of Bristol, Bristol, BS10 5 NB, UK; 4 NIHR Research Design Service South West, London, UK; 5 Nuffield Department of Orthopaedics (NOC), University of Oxford, Oxford, UK

**Keywords:** Total knee replacement, Prosthetic joint infection, Orthopaedic surgery, Feasibility, Equipoise, Recruitment, Pilot randomised controlled trial, Qualitative interviews

## Abstract

**Background:**

Total knee replacement surgery is common, with over 107,000 operations performed in the UK in 2019. After surgery, about 1% of patients develop a deep infection, known as a prosthetic joint infection. Two types of operations, one- or two-stage revision surgery, are routinely performed to treat the infection. Re-infection rates are similar, but there is uncertainty regarding longer-term outcomes for patients. The aim of this study was to establish the feasibility of conducting a future randomised controlled trial that will compare clinical and cost-effectiveness of one-stage versus two-stage revision knee surgery for prosthetic joint infection.

**Methods:**

Following eligibility screening, consenting patients took part in an audio-recorded consultation with their surgeon and were then randomised on a 1:1 allocation to one-stage or two-stage revision surgery. Patient-reported outcome measures were administered at baseline and 3 and 6 months postoperatively. Embedded qualitative work with patient participants and nonparticipants and with surgeons to understand the acceptability of trial processes and involvement was undertaken. Patient and public involvement and engagement activities were conducted throughout the study.

**Results:**

Of 136 patients screened, only 3 were randomised and had surgery as part of the study. Qualitative data were collected from the three participants, as well as from two eligible patients who declined participation and two who withdrew from participation after the initial patient-surgeon consultation. Five surgeons took part in qualitative interviews prior to study end.

**Conclusion:**

This study indicated that a larger randomised controlled trial evaluating one-stage versus two-stage revision knee surgery for prosthetic joint infection is not feasible with the current straightforward randomised controlled trial design. Future research needs to consider the most appropriate study design and methodology to address this important research question.

**Trial registration:**

No.: NCT04458961.

## Key messages regarding feasibility

- Key uncertainties regarding the feasibility of a randomised control trial of one- or two-stage revision surgery for knee PJI required investigation, including issues relating to surgical equipoise and recruitment.
- This study indicated that it was not feasible to recruit from the treatment centres included in the study, which tended to see complex revision cases who were either screened as ineligible or for whom there were specific reasons why one-stage or two-stage surgery would be better.
- Our study suggests a randomised controlled trial is not feasible, and that other study designs should be considered for addressing this important clinical question.

## Background

Over 107,000 primary total knee replacement (TKR) operations were performed in the UK in 2019 [1]. About 1.1% of patients with primary TKR develop a deep bacterial infection in the prosthetic joint within 2 years of surgery [2]. Previous studies have shown that prosthetic joint infection (PJI) and revision surgery to treat PJI impose heavy physical, social and psychological burdens on patients, often having a catastrophic impact on patients’ quality of life. Physically, patients may endure severe pain and prolonged periods of immobility resulting in an inability to participate in personal, work and leisure activities, leading to financial and employment difficulties and social isolation. Psychologically, patients have anxieties that infection may not be cured or that it may return, resulting in further surgery, long-term disability and possible amputation. Some patients report depression and suicidal thoughts [3, 4].

For most patients, PJI is managed with surgical revision involving extensive debridement and antibiotics followed by either one-stage or two-stage prosthetic replacement. With a two-stage revision, patients require two planned major surgeries. The first, to remove the infected prosthetic and an interim period of several months, allows targeted antibiotic treatment and monitoring of clinical status and inflammatory markers. This is followed by the second surgery to insert the new prosthesis. The alternative is a one-stage revision when removal, antibiotic treatment and insertion of new prosthetic all occur in a single operation. Although the one-stage surgery is complex and longer in duration, there is less overall surgery time and reduced length of hospital stay, which may lead to major cost savings and benefits for patients [5, 6]. Importantly, the literature does not clearly demonstrate a difference in infection eradication rates between the two techniques. In the most recent systematic review of longitudinal studies, rates of reinfection within 2 years of one-stage and two-stage revision were similar at 7.6% and 8.8%, respectively [7].

The recent INFORM randomised controlled trial (RCT) evaluated the clinical and cost-effectiveness of one- versus two-stage revision surgery for hip PJI [8]. The trial found that one-stage revision showed no superiority in patient-reported outcomes compared with two-stage revision at 18 months, although one-stage revision had a better outcome at 3 months, fewer intraoperative complications, and was cost effective (single-stage revision in hips cost £36,256 in comparison to £46,312 in the two-stage revision). However, there is no high-quality evidence to recommend a specific strategy, in terms of clinical or patient outcomes, for revision surgery for knee PJI. The need for a randomised evaluation has been widely recognised and warranted [7, 9–11] and aligns with 2 of the top 10 research priorities from the James Lind Alliance Priority Setting Partnership on revision knee replacement: ‘What is the best way to treat an infection of a knee replacement?’ and ‘Should revision surgery be done in one or two operations?’ [11]. However, prior to proceeding with a RCT, a feasibility study is necessary to address key uncertainties regarding whether a RCT is possible and explore how to optimise trial design and delivery. If patients are to be exposed to a major second operation with the potential mortality and morbidity associated with this, there needs to be evidence to support the benefit of this strategy. This evidence currently does not exist. At the outset of the study, two-stage surgery was the most commonly performed management of knee PJI within the UK, and several potential barriers to the success of the study were identified at the outset in the research protocol. These included the following:

- Surgeon equipoise may be lacking, when explaining the one- versus two-stage strategy.
- Patients may wish to pursue the more commonly performed two-stage surgery.
- Collecting the frequency of health and social care utilisation data is challenging.
- Trial recruitment is difficult to plan as the number and frequency of patients with PJI vary.
- Acutely unwell patients, requiring emergency surgery, may not be able to be recruited.
- The lack of study information in languages other than English may limit recruitment.
- Patients travelling from hospitals outside of the local areas may arrive with preconceived ideas of what treatment they require, as provided to them by their local surgeon, and hence may be unwilling to participate.

The aim of the MIKROBE study was to explore these challenges and determine the feasibility of progressing to a multicentre RCT comparing clinical and cost-effectiveness of one-stage versus two-stage revision knee surgery for PJI. Specific objectives were as follows:

- Evaluate the number of eligible patients.
- Establish recruitment rate.
- Assess feasibility of the process of explaining the study (explanation/recruitment/randomisation) between patients and surgeons (preoperative discussion about the study).
- Assess patients’ experiences (within 6 weeks after discharge following their definitive implant surgery) of recruitment and randomisation processes.
- Assess surgeons’ experiences postoperatively of recruitment and randomisation processes, as well as views about trial design.
- Assess and evaluate differences in study recruitment/attrition rates between study sites.
- Assess feasibility of performing one‐stage surgery.
- Assess adherence to the protocol at each site and between study sites.
- Assess mechanism of delivery and completion of pre‐ and post‐operative patient-reported outcome measures (PROMS).
- Assess ease of use of the healthcare resource utilisation in both surgical arms and estimate the cost of the intervention.
- Assess feasibility of collecting health-related quality-of-life data in both surgical arms.
- Identify out‐of‐pocket costs of the patient journey using both surgical arms.

## Methods

This pilot and feasibility study was conducted at four NHS trusts (study sites) which were high volume revision centres in the UK. Ethical approval was granted by Wales Research Ethics Committee 4 Wrexham (19//WA/0326, 13 Nov 2019, IRAS project reference: 272,334). The CONSORT extension to pilot and feasibility trials guidelines were adhered to in the reporting of this study. Four progression rules were established to indicate if a larger RCT would be feasible; these were as follows:

- Achieve target recruitment: Forty cases in 20 months.
- Establish the legitimacy and acceptability of the study protocol, including the acceptability of randomisation to both patients and surgeons.
- Prove case eligibility for both surgical approaches.
- Demonstrate adherence to the study protocol.

### Sample size

A sample size of 30 was considered sufficient to estimate standard deviations of continuous outcome measures [12]. To achieve 30 patients, it would be necessary to recruit 40 participants to allow for 25% attrition. Inviting 80 participants would offer an estimated 50% recruitment rate with an accuracy of 11 percentage points (95% *CI*: 39 to 61%). With 40 participants (20 per arm) recruited and an observed attrition rate of 25% in the pilot study (i.e. 10 patients), we could be 95% confident that the attrition estimate is accurate by ± 13 percentage points (i.e. 12 to 38%). In a 2-year period (2015 to 2017), there were 85 patients on the primary research site infection database. Of these, 30 met the inclusion criteria for the study (15 cases per year). Allowing that 20% of potential recruits would decline study entry, this left 12 cases per year from the research hospital. It was believed reasonable to assume a similar number from three additional participating centres. Based on this recruitment estimate, to achieve 40 cases, it was estimated that the sample required would be recruited in 20 months, during which time all eligible patients would be approached. Allowing 25% attrition, this would leave 30 cases completing the study. A sample greater than this would not substantially offer more accuracy in the standard deviation estimates.

### Participants

Patients with knee PJI were eligible for this study if they were considered suitable for either one-stage or two-stage revision surgery by their treating surgeon and the local multidisciplinary team. Inclusion criteria were as follows:

1. Age 18 years or older
2. A diagnosis of knee PJI according to the international consensus meeting criteria for infection [13]
3. Patients who had previously undergone debridement, antibiotics and implant retainment (DAIR) procedure or washout/biopsy of an infected TKR were still eligible to participate.
4. Revision surgery is indicated (one-stage or two-stage)
5. Intraoperatively, all cases must have adequate soft-tissue coverage post debridement.

### Exclusion criteria are as follows

1. Unable or unwilling to undergo either treatment
2. Lacking capacity to give written informed consent for participating in research
3. Refusal to consent to study for any reason
4. Re-revision of knee PJI if first revision was for infection
5. The presence of tuberculosis infection

### Interventions

#### One-stage revision TKR

These are removal of the infected prosthesis, extensive debridement of all involved tissues and implantation of the new prosthesis under the same setting, followed by targeted antibiotic treatment [14].

#### Two-stage revision TKR

Patients require two planned major surgeries and an interim period of several months. In the first operation, the prosthesis is removed, and the area extensively debrided. A spacer, typically made of cement, may be inserted to provide a temporary functioning joint and a source of local antibiotic delivery. The period between operations allows targeted antibiotic treatment whilst monitoring the clinical status and inflammatory markers. The duration, type and route of antibiotic treatment will be in line with local antibiotic guidelines at each participating centre. When the patient is clinically and biochemically clear of infection, the second stage consists of insertion of a new prosthesis [15].

### Recruitment and randomisation

Written informed consent was obtained from all participants before entering the study. Participants then completed baseline measures and attended the audio-recorded consultation with their surgeon. Following this consultation, randomisation was undertaken using a bespoke validated password-protected web-based site, developed and maintained by Exeter Clinical Trials Unit. Participants were randomly allocated in a 1:1 ratio to one-stage or two-stage revision surgery. Allocation concealment was ensured until patients were told the outcome of the randomisation.

### Quantitative data collection and analysis

Quantitative data collection included the following: study eligibility and recruitment rates, reasons for nonparticipation (at screening), PROMS completion rates, PROMS data comparison and a healthcare resource questionnaire adapted from the Client Service Receipt Inventory (CSRI) [16] which included a section on out-of-pocket costs. The EuroQoL (EQ- 5D- 5L) [17, 18] for measuring health-related quality of life for obtaining utilities for estimating quality-adjusted life years (QALYs) was also included. The statistical and economic analyses were to be performed blinded following pre-specified plans defined in the MIKROBE protocol, but these plans were subsequently adjusted to descriptive summaries due to the lack of recruitment to the study.

### Qualitative data collection and analysis

Acceptability of randomisation for patients and surgeons, and patients’ and surgeons’ experiences of surgery and trial participation, was explored qualitatively in six different ways: (1) brief interviews with patients declining to participate, (2) audio-recordings of the pre-surgery (and pre-randomisation) consultation between patients and surgeon, (3) brief interviews with patients following their definitive surgery, (4) brief interviews with patients withdrawing from the study, (5) brief interviews with surgeons after each surgery and (6) in-depth audio-recorded interviews with surgeons prior to the end of the study.

Consultations between patients and surgeons and end of study interviews with surgeons were audio recorded, transcribed, checked for accuracy and anonymised. The written notes of the ‘pro forma’ interviews with patients and surgeons were not transcribed and were securely transferred to the qualitative researcher. All qualitative data were imported into NVivo. The analysis of these data from the six possible data sources followed a framework approach which allowed for the inclusion of key concepts from the literature, alongside themes emerging from the data. Themes and theme summaries were initially developed to address the original research objectives. When a final set of themes and codes (analytical framework) had been developed agreed upon, incorporating researcher and PPIE perspectives, these were applied to all datasets with a second researcher checking a sample to ensure the data related to themes were interpreted consistently. Differences in interpretation were discussed within the wider qualitative research team.

### Patient public involvement and engagement

PPIE activities were undertaken with patients and stakeholders from the inception through to the dissemination of the research. The aims of the PPIE activities were to ensure acceptability of the study concept for patients, that patient burden through participation in the research was not onerous and that written patient-facing documents were concise, clear, understandable and reasonable. A PPIE member was invited to all TMG meetings and met with the research team during and at the end of the study to discuss the emergent findings, and their suggestions were incorporated into the discussion of results.

## Results

### Screening, recruitment and randomisation

**Fig. 1:**
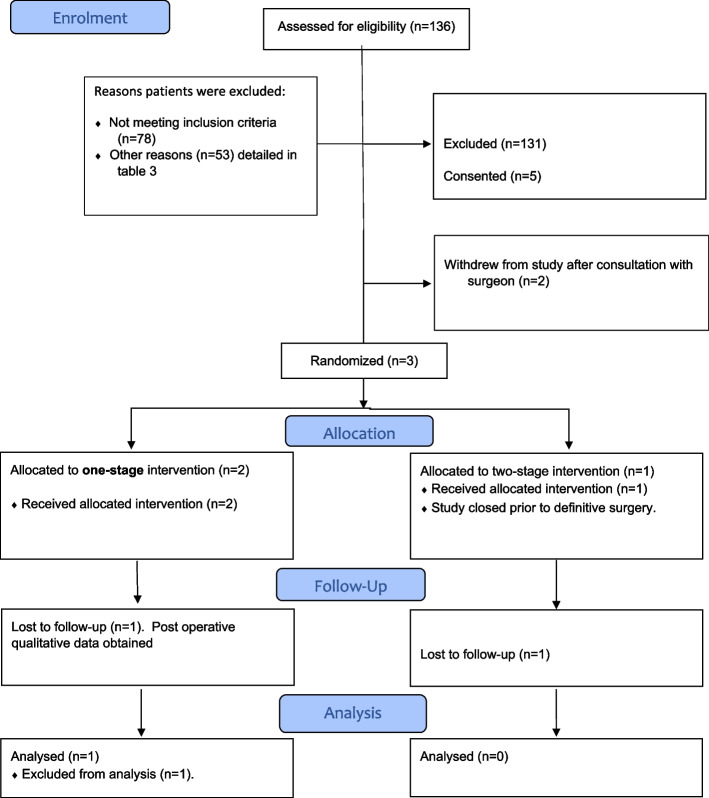
CONSORT flow diagram: MIKROBE study

Between July 2020 and October 2022, a total of 136 patients were screened across the 4 participating hospitals, of which only 5 patients consented to participate in the study (3.68%, 95% *CI*: 1.58 to 8.32%) (see Fig. 1, CONSORT flow diagram). During these 28 months, the study was paused for screening and recruitment for 8 months, from January 2021 until August 2021, due to the COVID- 19 pandemic. Two participants withdrew prior to randomisation after the surgeon–patient consultation. Three participants were randomised: two- to the one-stage surgery and one- to the two-stage surgery (see Table 1). Two eligible patients who declined to take part in the feasibility study agreed to be interviewed.

**Table 1:** Baseline characteristics for randomised participants (*n* = 3)

Participant characteristics	
Gender (male:female)	2:1
Age range (years)	67 to 84
BMI range	31 to 36
C-reactive protein range mg/L	101 to 124

Reasons for screening failure are provided in Table 2, with a breakdown of ‘other’ reasons in Table 3.

**Table 2:** Reasons for screening failure (*n* = 131)

Site	Reason for screening failure						
	**Unable or unwilling to undergo either treatment**	**Lacking capacity to consent to research**	**Refusal to consent to study (any reason)**	**Case does not meet the ICM criteria for infection**	**Re-revision for infection prosthetic knee infection**	**The presence of tuberculosis infection**	**‘Other’** **(see **Table 3**)**
1	18	2	3	10	20	0	45
2	2	0	3	7	11	1	9
3	0	0	0	1	0	0	1
4	0	0	0	0	0	0	0
Total (%)	20 (14.7%)	2 (1.47%)	6 (4.41%)	18 (13.24%)	31 (22.79%)	1 (0.74%)	55 (38.97%)

**Table 3:** Reasons described as ‘other’ in screening log

Reason given as ‘other’	Number excluded (%)
Native knee infection	**8** (5.9%)
Doubtful significance (not definite PJI)/uncertainty patient will have revision	**8** (5.9%)
Already had treatment/first stage at another hospital/had revision already	**7** (5.2%)
Complex case	**7** (5.2%)
Study recruitment closed before decision was made to revise or not	**5** (3.7%)
Not suitable for single-stage surgery/needed two-stage surgery	**5** (3.7%)
Trying to supress with antibiotics	**4** (2.9%)
No surgery pursued at all	**3** (2.2%)
Had debridement, antibiotics and implant retention and not for full revision	**2** (1.5%)
Significant vascular and skin issues so not eligible for one-stage surgery	**1** (0.7%)
MIKROBE study paused	**1** (0.7%)
Total femur replacement for fracture in infected TKR	**1** (0.7%)
Infection too extensive for two-stage surgery	**1** (0.74%)
**Total**	**55** (38.97%)

Table 4 provides an overview of screening, recruitment and randomisation at each site.

**Table 4:** Screening, recruitment and consent by study site

Site	Failed screen/screened/declined to participate	Consented (including to audio recording of consultation with surgeon)	Withdrawal	Randomised
Site 1	96/100	4	2	2
Site 2	33/33	0	0	0
Site 3	2/3	1	0	1
Site 4	0	0	0	0

### Quantitative data

All five patients who had provided informed consent completed baseline questionnaires. Post-operative questionnaires were completed by one participant at 3 and 6 months. One participant did not complete follow-up measures, and one participant had their second-stage surgery scheduled after the trial closed.

Due to the low number of cases, the planned statistical and economic analyses were inappropriate and replaced with the brief descriptive summaries (Tables 1, 2, 3 and 4).

### Qualitative data

Qualitative data were collected from the six data sources: (1) Two patients who had declined to take part (but were willing to be interviewed about their reasons for nonparticipation), (2) five consultations between patient and surgeon, (3) three patients who were randomised, (4) two patients who withdrew after the patient-surgeon consultation and prior to randomisation (but were willing to be interviewed), (5) two surgeons who were briefly interviewed after performing a definitive surgery for the trial (a third surgeon did perform a definitive second-stage surgery, but this occurred after trial closure) and (6) five surgeons involved in the trial who were interviewed near to the end of the trial (at least one surgeon from each of the four study sites). The qualitative findings are reported here in detail as these provided valuable insights into the issues related to the feasibility of conducting a future definitive RCT of these two types of surgery.

Four interrelated themes were identified from the six data sources. The themes and subthemes encompassed concepts from the literature, alongside findings emerging from the data (see also Tables 5 and 6). Theme 1 ‘Information and Communication’ related to what was provided to patients in a range of formats, from surgeons and other health professionals, during different timepoints of the care pathway. Information (provided both orally and in writing) was usually well received and appreciated by patients:

It [the patient information] was useful, quite wordy … I thought it was excellent to be quite honest …that [the surgeon consultation] was very enlightening…. (Participant 01 A)

**Table 5:** Themes and subthemes relating to MIKROBE study objectives: supporting quotes from patients

Themes and subthemes relating to MIKROBE objectives	Illustrative quotes from patients (including those remaining in the study, those declining to participate and those declining to participate having consented to a recorded consultation with their surgeon to hear more about the study)
**Theme: Information and communication** • Objective/outcome: Assessment of the process of explaining the study (explanation/recruitment/randomisation, equipoise) between patients and surgeons, to ensure it is suitable in the event of progressing to a definitive study • Objective/outcome: Analysis of reasons for patient’s nonparticipation and withdrawal • Objective/outcome: Analysis of the acceptability of randomisation to patients and surgeons, including recommendations for future trial design • Objective/outcome: Assessment of the feasibility of performing one-stage revision surgery	
**Evaluation or value of information provided**	*The information I was given told me everything I needed to know (Screened patient 63, declined to participate)*
**Sources and timepoints of information**	*I appreciate your time and everybody explaining me the study and the procedures (Screened patient 6, declined to participate)*
**Informed decision-making weighing up risk and choices based on information**	*Yeah so, you know, if my leg needs amputating, wake me up first. Make sure we’ve explored every option. By the same token, going into this surgery you know I kind of want to know what the route map is (Screened patient 54, withdrew after consultation with surgeon)*
**Theme: Recruitment and randomisation** • Objective/outcome: Assessment of the process of explaining the study (explanation/recruitment/randomisation, equipoise) between patients and surgeons, to ensure it is suitable in the event of progressing to a definitive study • Objective/outcome: Analysis of reasons for patient’s nonparticipation and withdrawal • Objective/outcome: Analysis of the acceptability of randomisation to patients and surgeons, as well as their thoughts about trial design • Objective/outcome: Assessment of the feasibility of performing one-stage revision surgery	
**Choice (wanting or not wanting choice) of surgical approach and whether or not to participate**	*So I think I’ve got the best of both options now. So the cunning plan I came up with is how about we aim for one stage surgery … but if I’m struggling with it during the operation… then you’ve obviously got the right to revert me back to a two stage operation which would give me relief from the anaesthetic in the short term… and he [the surgeon] was absolutely fine with that… (Screened patient 43, withdrew after consultation)*
**Computer ‘deciding’**	*I want quality of life and [the surgeon] knows me better than a computer … Decision is best left to the consultant. How does a computer decide what’s best for me? I am a bit old fashioned, a consultant will know me better than a computer will (Screened patient 63, declined to participate)*
**Complex patient needs** **‘Mr Average doesn’t exist’**	*I think the issue is that there isn’t an average patient. You know? Mr Average doesn’t exist. You know, you’ve got to overlay the individual circumstances onto the study… (Screened patient43, withdrew after consultation)*
**Research is a good thing**	*I am all for studies, anything that can improve things (Screened patient 63, declined to participate)*
’**Fed up’ with being ill**	*Really fed up and I just want it fixed (Screened patient 63 declined to participate)*
**Disempowered**	*I was in a bit of pain… trying to do two things at once was hard (Screened patient 54, declined to participate)*
**Theme: Equipoise, dissonance and challenging dogma** • Objective/outcome: Assessment of the process of explaining the study (explanation/recruitment/randomisation, equipoise) between patients and surgeons, to ensure it is suitable in the event of progressing to a definitive study	
**The average patient**	*Not just simply “well they’re having the type of surgery that we want to find out about”….Perfect client… no complications, nothing to, you know, consider outside of the box, you know? Mr Normal. He’d make the ideal client.. Outside of that, without me being rude, I think it just seemed too simplistic for me (Screened patient 43, withdrew after consultation)*
**Inclusion criteria**	*I think you need to think about, what are the things we think we need to look at to be able to quantify this patient should be a good fit. Because at the moment you’ve got it too broad… you need to make it a bit more focused (Screened patient 43, withdrew after consultation)*
**Patients weighing up risks**	*Well, I think we know what the risks are, but I just want to think about them a little bit, on my own … I want the single operation. However, I’d like my operation to be as risk adverse as possible… and the longer I’m under an anaesthetic, the more risky it becomes (Screened patient 43, withdrew after consultation)*
**Equipoise**	*… It didn’t make much difference to me as the knee was in quite a bad way anyway, so I thought why not just go for it. I was more than willing to do it (Participant 01 A, recruited to study, one stage surgery)*
**Dissonance**	*I think it’s worthwhile and yeah, anything that improves the outcomes of patients in orthopaedic surgeries, I’m all behind. You know, with the provisos I’ve given (Screen patient 43 withdrew after consultation)*
**Theme: Trial design** • *Objective/outcome: Assessment of the patient’s experience (within 6 weeks after discharge following their definitive implant surgery) of the process of surgery, recruitment and randomisation to ensure these processes are acceptable to them in the event of progressing to a definitive study (structured pro forma style questionnaire)*	
**Patient burden**	*Well it’s been a bit of a to-do. I was in hospital for three weeks, I was in intensive care and then I had COVID. I’ve been back and forth to the hospital a lot since. I don’t regret having it done though, it’s just been a long job. When you have your knee replaced you think it will be straightforward and the infections, they happen to someone else. When it does happen to you, well that’s a very difficult thing to live with (Participant 01 A, recruited to study, one stage surgery)*
**Cost drivers and considerations**	*With the other form [the questionnaires] it had all the trips and that.. but you haven’t allowed for being taken on these trips – my wife would have to drive me. Her loss of earnings as well as the cost of the fuel and parking. That I would need to be chauffeured around. So I got [the research nurse] to add that (Participant 01 A, recruited to study, one stage surgery)*

**Table 6:** End of study interviews with surgeons (*n* = 5) showing the four emerging themes and subthemes and include corresponding example quotes

Theme/subtheme	Illustrative quotes from end of study interview with surgeons involved in MIKROBE (*n* = 5)
**Theme: Information and communication**	
Patients identifying needs and preferences	*He knew he wanted to have it one go … so he actually, I think he pretty much told me “I know what I want, I am prepared to go through this process but I know what I want” (Surgeon 2)*
Share decision-making, shared decision-making based on information from a variety of sources	*Having an infection's a terrible thing to have and is very, very unpleasant and there's a pretty high failure rate. So personally I think it's really essential that the patient is involved in the decision, …It's their body, they can decide whatever they want … because when it fails, I think it's really important they felt, well, we decided together … (Surgeon 5)*
Patient equipoise	*… Within this study we’ve had experience of patients who are much more sort of involved in the decision making process and ultimately, after they’ve been given all the information, they’ve not found themselves in a position of equipoise ….The question of whether or not the patient has equipoise is very challenging and I think it is dependent on the patient, how much they want to know, you know (Surgeon 3)*
Surgeon credibility and expertise, patient scepticism and gaining trust	*And some people say, “oh well I don’t want to be a guinea pig”, and those are the ones that—that’s a nonstarter…. but by the time that that sort of conversation comes up and you’ve met them at least once, if not more, they kind of do trust you and so the credibility thing, and because they’re coming to a, you know, I suppose, a more reputable centre, and everyone’s said “we’re going to send you to this place and they’ll cure your infection” (Surgeon 5)*
**Theme: Recruitment and randomisation**	
Covid	*It was just everything seemed to change after Covid, …with number of previous surgeries, pre and post pandemic… (Surgeon 5)*
Wrong sites	*…I think looking through our data, I think the cases that came to us were far too complex to basically, I think in hindsight we were the wrong site for this sort of trial. (Surgeon 5)*
Increasingly complex (or acutely unwell) cases	*I think looking through our data, I think the cases that came to us were far too complex to basically (Surgeon 5)*
Changes or challenges with care pathways	*It’s always going to be a mixture of planned and ad-hoc work, … people are coming in in emergency settings are definitely eligible, the trick is trying to get to them before someone operates on them (Surgeon 9)*
**Theme: Equipoise, dissonance and challenging dogma**	
Equipoise — genuine uncertainty and living with uncertainty	*I suppose that's life as a clinician, isn't it, that you, at the end of the day, if you, [find] your equipoise disabling, you wouldn't be able to treat anybody, so, you know, at the end of the day, when you're treating someone, even if you're not sure what the best option is, you've got to go with what you and the patient think, with what you know … (Surgeon 9)* *I guess some of us have quite wide area, a wide area of patients where we have equipoise and some it's much more narrow and I suppose where that point is where, whether you can have an area where virtually every surgeon is uncertain about that question … (Surgeon 6)* *… Because I don’t know what the infection eradication rate is, I am, I mean in equipoise (Surgeon 5)*
Changing paradigms, challenging dogma	*Entirely incorrect the believe to think that two stage should result in lower chances of re-infection but difficult mindset to change (Surgeon 3)* *Even though, you know, there’s no science to back that up, I suppose that’s the, you know, that would be the conventional teaching and that’s the ingrained bias that you get (Surgeon 3)*
Infection eradication is key concern but many other factors to consider	*Better means I suppose getting a good infection eradication rate, so the primary aim of this operation is to get rid of the infection, the second aims are to have better function, … and minimise complications (Surgeon 5)*
Dissonance gut feeling and evidence required to change practice	*But you can't get away from that fact you're kind of, your gut wants to perform, do one or the other … at the time of randomisation, willing them to be one or the other, you know, well, I know, based on literature, *etc.,* I have equipoise, but you can't help, get away from that sort of experiential feeling about how you treat these cases because they're so complex you have to go with what you think's best, you can't sort of put them into an algorithm and say, this is best (Surgeon 6)*
Surgeons sometimes have preferences, which may not be based on evidence but on other things	*You know, sometimes it's probably subconscious things that you don't even appreciate you're thinking about, for example, you probably have biases that you didn't realise you had (…) as to whether they're a good host or a bad host, things in MDT that people say that sway you one way or the other (Surgeon 9)*
**Study design**	
Attitude to study, study design and study involvement	*Super keen to try and see if [it was possible] to get [a] randomised trial actually going, because it’s the ever present question amongst our community (Surgeon 5)*
Barriers and examples of recommendations	*If we maybe cast our web out wider we could each, but it would take a lot more hospitals and each hospital would contribute a small number I think. And then that’s, well that becomes a national trial which is really expensive (Surgeon 5)*
Considering other research designs such as cluster randomisation	*So certainly, I mean that would be a possibility, you know, one of the most experienced revision knee surgeons in the country does almost exclusively single stage, so you could recruit that unit to do single stage, […] I guess, very subjectively my view would be that surgeons would say they’ll be very happy to do either but actually you’d find it more straightforward to then find another site of a surgeon who was prepared to do a two stage one, so actually a cluster approach would potentially be… (Surgeon 3)*

Information and communication between surgeons (and other health professionals) and patients (and their families and carers) revealed variations in patients’ perception of trust in that information and impacted on patients’ decision-making related to trial participation:

…some people say, “oh well I don’t want to be a guinea pig”, and those are the ones that that’s a nonstarter. …then some patients say, “I just want you to decide for me what the best operationis” … emotionally that’s a bit more difficult, and you feel slightly more awkward saying to them “again, I don’t know which one’s better”, but I think, by the time that that sort of conversation comes up and you’ve met them at least once if not more, they kind of do trust you and so the credibility thing, and because they’re coming to a, you know, I suppose, a more reputable centre and everyone’s said “we’re going to send you this place and they’ll cure your infection”… (Surgeon 5)

Subthemes (see Table 5) were related to the patients’ evaluation of information and information timepoints and sources and how informed decision-making occurred, based on information.

Theme 2 ‘Recruitment and Randomisation’ reflects upon surgeons’ experiences of recruiting patients to the study and how the patient-surgeon consultation influenced subsequent decisions about proceeding to the randomisation. In one example, the surgeon describes misunderstandings (such as randomisation being misconstrued as ‘letting a computer decide’) that then impacted upon a patient’s decision to not consent to randomisation:

I don’t want a computer deciding it. I’ll do that myself. So, I think I’ve got the best of both options now. (Screened patient 43, withdrew after consultation)

The subthemes within this theme revealed patients’ perspectives regarding trial participation and the desire for shared decision-making, rather than necessarily handing the ‘decision’ over to the randomisation process. Some participants expressed a desire for choice, others described the burden of their condition and that they ‘just want it fixed’. There were complex biopsychosocial reasons underpinning patient decisions regarding study participation: the need to resolve the infection, beliefs about which surgery would result in the better outcomes given a particular infection or which surgery option was more manageable in terms of time off work or the impact on carers. Overall, patients generally recognised research to be a good thing, and three did proceed with randomisation, indicating their acceptance of the randomisation process.

The COVID lockdown restrictions created disruptions and changes to care pathways as routinely referred patients with uncomplicated knee PJI were not allowed to be transferred to the four high volume Revision centres were involved in the study. Instead, these specialist revision centres only received transferred patients with more complex profiles. The complexity of patient needs and the observation that ‘Mr Average Doesn’t Exist’ (PS54) were raised by a surgeon as one of the reasons why it became harder to recruit to the study:

If the patients were a little less complex it would have been a lot easier to recruit into this trial.… all the cases became slightly more complex, there was smatterings coming through that were like normal infections… (Surgeon 5)

Theme 3, ‘Equipoise, Dissonance, and Challenging Dogma’, revealed surgeon participants’ uncertainties and tensions regarding the best approach for treating knee PJI. To obtain high-quality evidence that addresses these issues, the surgeons needed to avoid being biased when recruiting patients, when discussing treatment options and when taking consent from patients to be randomised. Asking the surgeons to reflect upon their position of equipoise during this study was therefore a key focus of the in-depth interviews with the surgeons. They expressed a need for empirical evidence to challenge established surgical practices, norms and mindsets which meant one or other of the surgical options were preferred. Challenging this dogma was difficult as without ‘gold standard’ (e.g. RCT) evidence it was hard for surgeons to change. Furthermore, surgeons may have been trained to perform only one or other type of revision surgery which may have resulted in an understandable preference. However, surgical practices evolve and change over time, and the surgeons recognised the need for empirical evidence to support practice change; indeed, this need to challenge established views and practices was a key reason for undertaking the study.

It’s complex surgery, so they don’t want to try and risk it, and they [surgeons] have said to me, you know, “if you show me evidence that there’s no difference [In outcome measures] and that’s, one’s better than the other, I’ll change to the other one, but until then I’ll carry on doing what I’ve trained to do”. (Surgeon 5)

Patients and surgeons sometimes appeared to experience some dissonance when considering their options in relation to the two different surgical approaches. For example, patients viewed research and taking part in research positively and trusted their surgeon’s opinions and views but also described having preferences or ‘provisos’:

I think it’s worthwhile and yeah anything that improves the outcomes of patients in orthopaedic surgeries, I’m all behind. You know, with the provisos I’ve given you (Patient Screened patient 43, withdrew after consultation)

These preferences were grounded in the recognition that there were sometimes clear indications that one type of surgery would be preferable to the other, including microbiological factors, comorbidities, infection in other parts of the body, local factors (such as soft tissue, wounds or sinus, bone loss), psychosocial factors and service constraints such as space on operating lists, particularly during the Covid and immediately post-Covid era.

The patients are so multifactorial that actually, the fact that one thing might be better than that thing, but it might be influenced by so many more factors than just the piece of metal you’re putting in, you know, there’s the patient preference there’s the pain levels, there’s the psychological factors …So even for any type of sort of surgery it’s quite difficult and that’s why it’s so difficult to do a really good study (Surgeon2)

It was clear that whilst surgeons recognised the significant extra burden of two-stage surgery (particularly for patients but also in terms of NHS resources), at the same time, they had personal inclinations regarding which surgical approach should be taken:

You can't get away from that fact you're kind of, your gut wants to perform, do one or the other (Surgeon 5)

The ‘gut feeling’ of which is the best treatment for a particular patient might suggests a lack of equipoise and reflects the dissonance felt by surgeons as they recognised the lack of empirical evidence to underpin decisions.

With the 2 stages I think we can be absolutely clear that there is no more infection in the cavity before putting the other implant (Surgeon 6)

However, it was also clear that eligible patients were also not necessarily in a state of equipoise: two participants having one-stage surgery expressed a preference for this, and one patient, who did not proceed to consent to randomisation, expressed a clear preference for two-stage surgery.

Theme 4 collates views about the ‘Trial Design’ and recommendations for any future study. Suggestions from surgeons, patients and PPIE representatives included modifications to trial design (for example cluster randomisation, platform and pragmatic trial designs):

If you go for a sort of platform type design where there are different strands to it, I think you’ll obviously recruit a lot more patients but we’ll see them going into the easier strands of the study, you know, i.e. should they have revision versus a DAIR, for example, that’s an easier study to put patients into than going sort of all out for a single versus two stage but at least you’ll, you know, you’ll answer, you’ll probably answer more questions at the end of the day, even if it’s not that specific (Surgeon 3)

Other suggestions included a nationwide review of surgical practices, as well as modifications to inclusion criteria (for both patients and the surgical centres), screening and recruitment processes. For example, by the end of the study, it was clear that many more patients than originally anticipated were not suitable:

Probably the thing I think we didn’t think about was that actually most of them come through acutely […] When they come in acutely, slightly unwell and they’ve got systematic sepsis, then they’re not really suitable for the study if they’re septic. (Surgeon 2)

Additional recommendations were for further qualitative research to explore the relative burdens of the two types of surgery for patients and whether surgeon equipoise could ever be achieved within the clinical community.

## Discussion

The criteria justifying progression to a larger, definitive RCT, including the recruitment of 40 participants in 20 months, were not met. One reason was that far fewer patients were found to be eligible at the study sites than anticipated. Although one study site screened relatively large numbers of cases, few were eligible, and thus, conversion to recruitment was low. A relatively small number of screening fails due to patients being unwilling to be randomised; the qualitative component of the study confirmed how the complexity of patients’ needs, post-COVID- 19 changes to referrals to high volume specialist revision centres and the consequent developments in surgical practice at local hospitals (where more surgeons were now practicing one-stage surgery) all impacted on recruitment. The COVID pandemic had led to elective surgery cancellations during lockdowns which persisted afterwards due to staff shortages and winter pressures. As primary TKRs were not performed for nearly a year, the predicted number of patients at risk of developing PJI markedly decreased, further reducing the pool available for recruitment. By the study end, surgeons commented that far fewer patients than anticipated were equally suitable for either type of surgery. A recent systematic review and meta-analysis of single- versus two-stage revision for periprosthetic joint infection called for a further prospective RCTs whilst also highlighting the highly individual nature of the varied factors that needed to be considered carefully on a patient-by-patient basis [19]. In our study, recruitment was further hindered by lack of time to approach patients in emergency care settings who were proceeding to surgery. In addition, diagnosis of PJI is challenging, and, in the absence of positive tissue cultures or a sinus communicating with the joint, clinicians were reliant on multiple clinical tests, which cannot provide a definitive diagnosis. These factors all contributed to a high number of patients being screened for possible infection but ultimately not fulfilling the criteria for study inclusion. This suggests that a review of screening criteria in any future study is warranted. The low recruitment numbers prevented assessment of the three remaining progression rules.

In addition to the progression criteria, a number of challenges to feasibility, which were highlighted at the study inception, were explored qualitatively. A lack of surgeon equipoise was anticipated, yet surgeons felt they were in equipoise for the patients who were recruited to the study. In contrast, the screening log suggested that surgeons were in equipoise for far fewer patients than originally estimated, and this was primarily due to the complexity of patient cases; it was apparent that patients were also not always in a position of equipoise regarding the concepts of RCTs. A further challenge was the variability in throughput of patients with PJI, making it difficult to predict and plan recruitment numbers. This anticipated variability was dramatically affected by COVID- 19 which created a fundamental and enduring change in the care pathway: the collapse of routine primary TKRs meant that the usual small numbers of PJI (around 1%) were also diminished, and some who did come for surgery were seen in emergency care settings with little time available to create a screening opportunity.

Further exploration as to why patients may wish to pursue two-stage surgery, the impact of study information only being available in English and the concern that patients from hospitals outside of the local areas may have had preconceived ideas of what treatment they required were issues that could not be fully addressed because of the low recruitment numbers. No evidence emerged to suggest that patients would prefer two-stage surgery or that one-stage was unacceptable; rather, it was specific patient needs meant that one or other was preferable, and, importantly, that there was a routinely available surgical offer. This clinical landscape differed from that of the recently completed INFORM trial [8], where patients were routinely only offered two-stage surgery, meaning that participation in the INFORM RCT allowed access to an alternative treatment that was not otherwise available to patients.

In line with previous research [3], surgeons highlighted need for evidence to support their decision-making regarding surgery options. They acknowledged the established view that two-stage surgery which offered the best chance of infection eradication could be ‘entirely untrue’ (S03) as there was no supporting empirical evidence. These established views were nonetheless powerful agents in decision-making and were regarded as dogma which would be hard to change. However, some surgeons were changing their views and/or their practice, especially in local centres due to the pandemic’s influence on referral pathways. Surgeons also recognised the well-documented and extreme burden of PJI and its subsequent treatment for patients [3, 4, 20–22], and that two-stage surgery may prolong burden for patients and result in additional NHS costs. The emerging theme ‘Equipoise, Dissonance and Challenging Dogma’ reflected surgeons’ desire to minimise the burden for patients, and not to ‘over-treat’ patients because of established dogma, yet at the same time avoid the catastrophic outcome of not eradicating infection. This contributed to cognitive dissonance [23] for some surgeons, and similar decision-making discomfort was also described by some patients. Patients were required to take on board and make sense of highly complex information about their condition; they placed high levels of trust in their surgeon and at the same time wanted to be involved in decisions made about their care, and they ‘did not want a computer to decide’ but did often feel that their ‘surgeon knows best’, even when their surgeon expressed being in a position of equipoise. Similar to other studies [24], patients generally saw research as a good thing and were thus motivated to participate. Yet, patients exhibited motivated reasoning [22] which also influenced their decision-making, and they weighed up costs and benefits of participating, resulting in conditional altruism [24] that ultimately influenced their decisions both whether to take part initially and whether to stay in the study after consulting with their surgeon [24].

The impact of the COVID pandemic was both immediate and enduring and should not be underestimated in terms of how it affected the patient volume, pathways and recruitment. However, the findings from this feasibility study shows that a simple definitive RCT comparing one- or two-stage revision TKR surgery for PJI, using the methods and processes we employed in this study, would not be feasible. It may be that the simple RCT design is not the most suitable method for obtaining empirical evidence for complex orthopaedic surgery. Nonetheless, surgeons continue to recognise the importance of the original research question intended to be addressed by MIKROBE, and a platform type trial design may be more appropriate as these allow multiple questions to be evaluated simultaneously and evaluate interaction between different treatment options to achieve the goal of determining the optimal combination of treatments. MIKROBE’s qualitative findings indicated that patient choice and ability to share in decision-making were of key importance, and future research may need to build this into study designs. Concepts such as cognitive dissonance and motivated reasoning may help to provide further insights into how knowledge sources are used by both patients and clinicians in decision-making and in the processing of uncertain and complex information. The need to recruit participants requiring more ‘straightforward’ revision surgery, possibly from a larger number of centres, and the necessity to incorporate patients’ needs and preferences into the decision -making for surgery, were highlighted as key areas for consideration in future research.

### Strengths and limitations

It was recognised early on that recruitment was significantly lower than anticipated and impacted by COVID- 19 restrictions. The addition of two research sites did not improve recruitment. The qualitative analysis drew upon a range of data sources, and the resultant convergence of findings is a strength. The qualitative enquiry addressed the research objectives and provided insights that will be useful when designing future research. The small numbers of participants are characteristic of in-depth qualitative enquiry, and findings will be transferable to other research, but these should not be considered generalisable.

## Conclusions

The two research priorities identified by the James Lind Alliance Priority Setting Partnership on revision knee replacement: ‘What is the best way to treat an infection of a knee replacement?’ and ‘Should revision surgery be done in one or two operations?’ remain of the upmost clinical importance, to both patients and clinicians. Conventional national RCTs for such a group of patients have proved not possible. Experience from the INFORM study indicates that a multinational study involving 15 centres from across Europe was required to achieve their recruitment targets. Furthermore, it is important to remember that whilst the INFORM study was examining single- versus two-stage revision for infection, this was in relation to the hip and not the knee. Whilst hip and knee arthroplasty have many themes in common they are fundamentally different operations with contrasting patient, microbiome and technical characteristics.

Amongst the international clinical orthopaedic community, there is consensus that this key and complex problem, which causes such severe patient suffering, requires a multinational approach. An international platform trial could individually address pieces of this multifaceted jigsaw, as attempting this in one single trial is too simplistic an approach.

## Data Availability

Not enough quantitative data were gathered to warrant any data sharing. Qualitative data that support the findings of this study are not openly available due to reasons of sensitivity but can be made available from the corresponding author upon reasonable request.
